# A Sample Preparation Method for the Simultaneous Profiling of Signaling Lipids and Polar Metabolites in Small Quantities of Muscle Tissues from a Mouse Model for Sarcopenia

**DOI:** 10.3390/metabo12080742

**Published:** 2022-08-12

**Authors:** Yupeng He, Marlien van Mever, Wei Yang, Luojiao Huang, Rawi Ramautar, Yvonne Rijksen, Wilbert P. Vermeij, Jan H. J. Hoeijmakers, Amy C. Harms, Peter W. Lindenburg, Thomas Hankemeier

**Affiliations:** 1 Metabolomics and Analytics Centre, Leiden Academic Centre for Drug Research, Faculty of Science, Leiden University, Einsteinweg 55, 2333 CC Leiden, The Netherlands; 2 Princess Máxima Center for Pediatric Oncology, 3584 CS Utrecht, The Netherlands; 3 Oncode Institute, 3521 AL Utrecht, The Netherlands; 4 Department of Molecular Genetics, Erasmus MC Cancer Institute, Erasmus University Medical Center Rotterdam, 3015 GD Rotterdam, The Netherlands; 5 Institute for Genome Stability in Aging and Disease, Cologne Excellence Cluster for Cellular Stress Responses in Aging-Associated Diseases (CECAD), University of Cologne, 50931 Cologne, Germany; 6 Research Group Metabolomics, Leiden Center for Applied Bioscience, University of Applied Sciences Leiden, 2333 CK Leiden, The Netherlands

**Keywords:** metabolomics extraction, signaling lipids, polar metabolites, muscle tissue, muscle ageing and sarcopenia

## Abstract

The metabolic profiling of a wide range of chemical classes relevant to understanding sarcopenia under conditions in which sample availability is limited, e.g., from mouse models, small muscles, or muscle biopsies, is desired. Several existing metabolomics platforms that include diverse classes of signaling lipids, energy metabolites, and amino acids and amines would be informative for suspected biochemical pathways involved in sarcopenia. The sample limitation requires an optimized sample preparation method with minimal losses during isolation and handling and maximal accuracy and reproducibility. Here, two developed sample preparation methods, BuOH-MTBE-Water (BMW) and BuOH-MTBE-More-Water (BMMW), were evaluated and compared with previously reported methods, Bligh-Dyer (BD) and BuOH-MTBE-Citrate (BMC), for their suitability for these classes. The most optimal extraction was found to be the BMMW method, with the highest extraction recovery of 63% for the signaling lipids and 81% for polar metabolites, and an acceptable matrix effect (close to 1.0) for all metabolites of interest. The BMMW method was applied on muscle tissues as small as 5 mg (dry weight) from the well-characterized, prematurely aging, DNA repair-deficient *Ercc1^∆/−^* mouse mutant exhibiting multiple–morbidities, including sarcopenia. We successfully detected 109 lipids and 62 polar targeted metabolites. We further investigated whether fast muscle tissue isolation is necessary for mouse sarcopenia studies. A muscle isolation procedure involving 15 min at room temperature revealed a subset of metabolites to be unstable; hence, fast sample isolation is critical, especially for more oxidative muscles. Therefore, BMMW and fast muscle tissue isolation are recommended for future sarcopenia studies. This research provides a sensitive sample preparation method for the simultaneous extraction of non-polar and polar metabolites from limited amounts of muscle tissue, supplies a stable mouse muscle tissue collection method, and methodologically supports future metabolomic mechanistic studies of sarcopenia.

## 1. Introduction

Sarcopenia is characterized by the age-related loss of muscle mass and function, constitutes a major health problem, and is associated with a high loss of quality of life [1,2]. Globally, 11–50% of those aged 80 or above suffer from sarcopenia [3], and this number is increasing with the rapid growth of the ageing population, thereby creating an enormous socioeconomic and health care burden. The molecular mechanisms underlying sarcopenia are still not well understood and effective medication is lacking [4]. Metabolomics is a powerful approach for obtaining molecular insight into complex diseases and for the discovery of disease biomarkers [5]. Previous muscle function metabolomics studies revealed that dysregulation of signaling lipids (i.e., oxylipins, free fatty acids, oxidative stress markers) [6,7,8], energy metabolites (i.e., ATP, citrate, pyruvate) [9,10,11], and amino acids and amines [10,12,13] were highly associated with weak muscle contractile function. Therefore, a systematic metabolomics mechanistic study of these non-polar (signaling lipids) and polar (energy metabolites, amino acids and amines) metabolites is needed for understanding the biochemistry behind sarcopenia and for the identification of biomarkers for the diagnosis, prevention, and treatment of sarcopenia. Mice deficient in the DNA excision-repair gene, *Ercc1* (*Ercc1^∆/−^*), show numerous age-related pathologies and accelerated ageing features [14,15,16], and are widely used in the studies of ageing and age-related diseases, including muscle wasting and sarcopenia [17,18,19,20]. Moreover, this mouse mutant is an excellent model for several rare, but very severe progeroid human DNA repair syndromes, including Cockayne syndrome, xeroderma pigmentosum, Fanconi anemia, and XFE1 syndrome [21,22,23]. As the *Ercc1^∆/−^* mice exhibit early cessation of growth, only small amounts (i.e., 5–50 mg dry weight) of (skeletal) muscle can be collected, necessitating the development of a single sensitive, reproducible sample preparation method suitable for analysis by multiple metabolomics platforms, thereby allowing for the analysis of non-polar and polar metabolites.

The Bligh and Dyer (BD) method is a traditional sample preparation method for the extraction of non-polar and polar components, which is able to non-selectively extract a wide range of metabolites [24,25]. Medina et al. evaluated sample extraction methods with isopropanol and 1-butanol:methanol for simultaneous extraction of 584 non-polar and 116 polar metabolites; however, the method mainly focused on the metabolome analysis of human plasma samples and some of our targeted signaling lipids—oxylipins and bile acids were not covered [26]. Löfgren developed an automated butanol:methanol extraction method for lipids, however, the method mainly focused on the plasma lipid classes, i.e., cholesterol, triacylglycerol, phosphatidylcholine, sphingomyelin, and lyso-phospholipids [27]. BuOH-MTBE-Citrate (BMC) is a sensitive sample preparation method for sample limited applications; Di Zazzo et al. applied this method for the analysis of oxylipins, oxidative stress markers, endocannabinoids, and bile acids for ocular surface cicatrizing conjunctivitis, and identified 9S-hydroxy octadecatrienoic acid (9S-HOTrE) and 5-hydroxy eicosapentaenoic acid (5-HEPE) as potential diagnostic biomarker candidates. However, the performance of BMC on a small amount of muscle tissues still remains unknown, and because of the addition of a non-volatile (citric acid/phosphate) buffer, the extracted aqueous phase was not compatible with mass spectrometric detection [28].

In this work, we report the development of a sample preparation method that allows for the simultaneous extraction of targeted non-polar and polar metabolites from biomass-limited mouse muscle tissues (i.e., 5–50 mg dry weight). With this approach, we would like to obtain more insight into the etiology of sarcopenia using a metabolomics approach. For this purpose, two extraction methods based on BMC [28] were developed and compared with Di Zazzo et al.’s BMC [28] and BD methods [29]. The optimal method with the highest extraction recovery and acceptable matrix effect was applied to muscle tissues of *Ercc1^∆/−^* mice to study the effect of the muscle tissue isolation speed on metabolite stability. Overall, this work yielded a sensitive sample preparation method for the simultaneous extraction of non-polar and polar metabolites from limited amounts of muscle tissues, supplied a reference method for an existing sarcopenia samples collection, and methodologically supports the metabolomic analysis of sarcopenia.

## 2. Materials and Methods

### 2.1. Chemicals

Methanol and chloroform were purchased from Biosolve Chimime SARL (Dieuze, France). The 1-butanol was purchased from Acros Organics (Geel, Belgium). Butylated hydroxytoluene (BHT) and methyl tert-butyl ether (MTBE), and citric acid and sodium dihydrogen phosphate dehydrate were obtained from Sigma-Aldrich (Steinheim, Germany). MilliQ water was obtained from a Millipore high-purity water dispenser (Billerica, MA, USA). All solvents were HPLC grade or higher.

For internal standards (ISTDs), deuterium-, carbon-, and/or nitrogen-labelled metabolites were used. Labelled oxylipins, fatty acids, and endocannabinoids ISTDs were acquired from Cayman Chemicals (Ann Arbor, MI, USA). Labelled lysophospholipids, sphingolipids, and bile acid and steroid ISTDs were ordered from Avanti Polar Lipids (Alabaster, AL, USA). Labelled amino acids and amine ISTDs were ordered from Cambridge Isotope Laboratories (Andover, MA, USA), and labelled ATP, AMP, and UTP were purchased from Sigma-Aldrich (Steinheim, Germany).

### 2.2. ISTDs Preparation

For lipid ISTDs, the stock solution was prepared in MeOH in a stated concentration (Table S1) containing 0.4 mg/mL BHT. This includes the classes of oxylipins, fatty acids, endocannabinoids, bile acids and steroids, lysophospholipids, and sphingolipids. For the stock solution of amino acids and amine ISTDs, 9 kinds of ISTDs (Table S2) were prepared in MilliQ water with a concentration of 0.5 mg/mL. Stock solutions of ATP (^13^C_10_,^15^N_5_), AMP (^13^C_10_,^15^N_5_), and UTP (^13^C_9_,^15^N_2_) were prepared in MilliQ water at 10 mg/mL (Table S3).

### 2.3. Muscle Samples

The development and evaluation of extraction methods were performed on pig muscle tissues that serve as a uniform source for multiple experiments and as a surrogate for mouse tissue, which was only available in scarce quantities. The pig muscle tissue was stored at −80 °C before extraction. Muscle tissue from mice deficient in the DNA excision-repair gene *Ercc1* (*Ercc1^∆/−^*) was utilized for the study of effect of sample isolation speed on metabolite stability for sarcopenia. The generation and characterization of *Ercc1^∆/−^* mice is described in [15,16,20]. Three kinds of muscle types, gastrocnemius + soleus (Gas + Sol), quadriceps (Quadr), and extensor digitorum longus + tibialis anterior (EDL + TA), were collected at the animal facility of the Erasmus Medical Center, Rotterdam, Netherlands. All above experiments were performed in accordance with the Principles of Laboratory Animal Care and with the guidelines approved by the Dutch Ethical Committee (permit Nos. 139-12-13 and 139-12-18) in full accordance with European legislation.

Fast and delayed (15-min delayed) muscle tissue collection procedures were applied to study the effects of sample isolation speed on metabolite stability. Briefly, mice were anaesthetized using CO_2_. For fast sample isolation, a large piece of Quadr tissue was dissected immediately and rapidly frozen in liquid nitrogen, and EDL + TA and Gas + Sol tissue were carefully isolated as described in [30]. Following dissection, the muscles were immediately frozen in liquid-nitrogen-cooled isopentane and stored at −80 °C [19]. For delayed sample isolation, the Quadr, EDL + TA, and Gas + Sol tissues from the other hind leg of the same mouse were kept for 15 min at room temperature, then were isolated and frozen as described above for the fast isolation. All samples were stored at −80 °C until analysis.

### 2.4. Extraction Methods

For the development of an extraction method yielding high extraction efficiency for both polar metabolites and signaling lipids, four extraction methods were compared and evaluated using pig muscle tissues, i.e., the Bligh-Dyer (BD), BuOH-MTBE-Citrate (BMC), BuOH-MTBE-Water (BMW), and BuOH-MTBE-more-Water (BMMW) extraction methods. Thirty mg (±20%) of frozen wet pig muscle tissue was lyophilized in a VaCo I freeze-dryer (Zirbus, Bad Grund, Germany; connected to a E2M12 high vacuum pump, Edwards, Crawley, England) for 24 h and weighed. To homogenize muscle tissues thoroughly, a dry-homogenization method was used by adding 100 mg (±10%) of zirconium oxide beads (0.5 mm; Next Advance, Averill Park, NY, USA) to the freeze-dried tissue, and homogenized in a Bullet Blender (BBX24; Next Advance, Averill Park, NY, USA) for 15 min at speed 9 [29]. Labelled ISTDs (10 µL amino acids & amines, 10 µL ATP & AMP & UTP, 10 µL lipids stock solution) were spiked in the muscle samples before and after extraction for the evaluation of the four extraction methods.

#### 2.4.1. Bligh-Dyer Extraction (BD)

A previously reported Bligh-Dyer extraction was utilized for the polar and non-polar analyte extraction [29]. Briefly, 400 µL of cold MeOH and 125 µL of cold MilliQ water were added to the muscle tissues and homogenized by using the Bullet Blender for 15 min at speed 9. Then, 450 µL of homogenate was transferred to a new tube after centrifugation (500× *g*, 5 min, 4 °C), and vortexed with cold chloroform (450 µL), water (250 µL), and MeOH (50 µL) for 2 min. The samples were next left on ice for 10 min to partition, and centrifuged (2000× *g*, 10 min, 4 °C) to obtain a clear biphasic mixture. The 500 µL of upper aqueous/polar phase and 400 µL of lower organic/non-polar phase were collected separately by using positive-displacement Microman pipettes (Gilson, Middleton, WI, USA) without disturbing the layer between both phases. These were then evaporated in a SpeedVac Vacuum concentrator (Thermo Savant SC210A, Waltham, MA, USA) and reconstituted in 50 µL of MeOH for the organic phase and 100 µL of 50%-MeOH–50%-MilliQ water for the aqueous phase.

#### 2.4.2. BuOH-MTBE-Citrate Extraction (BMC)

A reported lipid extraction method [28], BuOH-MTBE-Citrate extraction (BMC), was tested for the muscle samples. In this method, 5 µL of antioxidant solution (0.4 mg/mL BHT:EDTA = 1:1), 150 µL of 0.2 M citric acid-0.4 M disodium hydrogen phosphate buffer at pH 4.5, and 1 mL of extraction solution (BuOH: MTBE = 1:1, *v*/*v*) were added to all samples and allowed to settle on ice for 20 min before homogenization in the Bullet Blender for 15 min at speed 9. Then, the homogenized samples were centrifuged (2000× *g*, 4 °C) for 10 min, and 900 µL of the upper organic phase was collected, evaporated, and reconstituted using the same method described in 3.4.1 BD method.

#### 2.4.3. BuOH-MTBE-Water Extraction (BMW)

The extraction procedure for the BMW method is similar to the BMC method, but the 150 µL of citric acid/phosphate buffer was replaced with 150 µL of cold MilliQ water. After collection of the upper organic phase, 500 µL more of ice-cold MilliQ water was added to more easily collect the lower aqueous phase. After vortexing and centrifugation at 2000× *g* at 4 °C for 10 min, 350 µL of the lower aqueous phase was then collected.

#### 2.4.4. BuOH-MTBE-More-Water Extraction (BMMW)

A larger aqueous phase volume (400 µL of cold MilliQ water) was utilized in the BMMW method instead of the 150 µL of cold MilliQ water used in the BMW method. Two-hundred µL of the lower aqueous phase was directly collected after collection of the upper organic phase.

### 2.5. LC/CE-MS Quality Control

Some extra extracted pig muscle tissues were pooled together as quality control (QC) samples. A QC sample was injected once each 6–8 samples to evaluate and correct for changes in the sensitivity of the instruments. The metabolites with a relative standard deviation (RSD) of quality control (QC) samples less than 30% were used for statistical analysis.

#### 2.5.1. Lipid Metabolite Analysis

The signaling lipid metabolites were measured according to a validated ultra-performance liquid chromatography tandem mass spectrometry (UPLC-MS/MS) method in our lab [28]. Briefly, each sample was measured with two complementary reverse phase methods using mobile phases with a different pH.

The low-pH run utilized an Acquity BEH C18 column (50 × 2.1 mm, 1.7 μm; Waters, Milford, USA) on a Shimadzu LC-30AD (Kyoto, Japan) hyphenated to a SCIEX Q-Trap 6500+ (Framingham, MA, USA). Separations were performed using three mobile phases: (A) water with 0.1% acetic acid; (B) ACN: MeOH (9:1, *v*/*v*) with 0.1% acetic acid; (C) Isopropanol with 0.1% acetic acid at 40 °C at a flow rate of 0.7 mL/min. The 16-min run used the following gradient: start with 20% B and 1% C; B was increased to 85% between 0.75 and 14 min and C was increased to 15% between 11 and 14 min; and the condition held for 0.5 min prior to column re-equilibration at the starting conditions from 14.8 to 16 min. Data were acquired using Sciex Analyst software (Version 1.7, Framingham, MA, USA) and peak integration used Sciex OS (Version 1.4.0, Framingham, MA, USA).

The high-pH run used a Kinetex^®^ Core-Shell EVO 100 Å C18 column (50 × 2.1 mm, 1.8 μm; Phemomenex, Torrance, CA, USA) on a Shimadzu LCMS-8060 system (Shimadzu, Kyoto, Japan). Separations used mobile phases: (A) 5% ACN with 2 mM ammonium acetate and 0.1% ammonium hydroxide and (B) 95% ACN with 2 mM ammonium acetate and 0.1% ammonium hydroxide at 40 °C at a flow rate of 0.6 mL/min. The gradient started with 1% B; B was increased to 100% from 0.7 to 7.7 min; and 100% B held for 0.75 min prior to re-equilibration at the starting conditions between 8.75 and 11 min. Multiple reaction monitoring (MRM) was utilized in MS/MS acquisition in both the positive and negative electrospray ionization mode with polarity switching. Data were acquired and peaks integrated using LabSolutions (Version 5.97 SP1, Shimadzu, Kyoto, Japan).

#### 2.5.2. Energy Metabolites Analysis

The energy metabolites were analyzed using a hydrophilic interaction liquid chromatography (HILIC) mass spectrometry platform [31]. Briefly, a SeQuant ZIC-cHILIC column (PEEK 100 × 2.1 mm, 3.0 μm particle size; Merck KGaA, Darmstadt, Germany) was used on a Waters UPLC (AcquityTM, Milford, MA, USA) coupled with a Sciex MS (Triple-TOF 5600+, Framingham, MA, USA). The separation method used mobile phases: (A) 90% ACN with 5 mM ammonium acetate at pH 6.8 and (B) 10% ACN with 5 mM ammonium acetate at pH 6.8, at a flow rate of 0.25 mL/min at 30 °C. The gradient method was: 100% A for 2 min; ramping 3–20 min to 60% A; ramping 20–20.1 to 100% A; and re-equilibrated to 35 min with 100% A. The MS data were acquired at a full scan range of 50–900 *m*/*z* in the negative ionization mode with curtain gas measured at 39.3 psi, source temperature at 400 °C, and ion source voltage at 4.64 kV by Sciex Analyst (Version 1.7, Framingham, MA, USA), and the peaks were integrated using MultiQuant (Version 3.0.1, Sciex, Framingham, MA, USA).

#### 2.5.3. Amino Acids and Amines Analysis

The amino acids and amines were analyzed by a sheath-liquid Agilent 7100 capillary electrophoresis (CE) system, coupled to an Agilent mass spectrometer (TOF 6230, Waldbronn, Germany), and acquired by MassHunter Data Acquisition (Version B.05.01, Agilent, Santa Clara, CA, USA). Fused-silica capillaries (BGB Analytik, Harderwijk, Netherlands) with a total length of 70 cm and an internal diameter of 50 µm were utilized. The CE separation voltage was 30 kV, and 10% acetic acid in water was used as a background electrolyte (BGE) solution. The sheath-liquid, a mixture of water and isopropanol (50:50, *v*/*v*) containing 0.03% acetic acid, was delivered at a flow rate of 3 µL/min by an Agilent 1260 Infinity Isocratic Pump (Waldbronn, Germany). The nebulizer gas was set to 0 psi, the sheath gas flow rate was set at 11 L/min, and the sheath gas temperature was set at 100 °C. The ESI capillary voltage was set at 5500 V. The fragmentor and skimmer voltages were 150 V and 50 V, respectively. MS data were acquired in the positive ion mode between 50 and 1000 *m*/*z* with an acquisition rate of 1.5 spectra/s [32]. The amino acids and amines peaks were integrated using MassHunter Quantitative Analysis (Version 05.02, Agilent, Santa Clara, CA, USA).

### 2.6. Data Analysis

For metabolites for which QC samples had an RSD less than 30%, the response ratios were corrected by QC response ratio and further normalized by the muscle tissue dry weight. For metabolites that can be measured by multiple platforms, i.e., amino acids/amines (which can be measured by the HILIC and CE methods in Section 2.5.2 and Section 2.5.3, respectively) and some fatty acids (which can be measured by both low- and high-pH lipid platforms), the method with the smaller QC RSD was utilized (Tables S4 and S5).

For the comparison and evaluation of the developed extraction methods, the extraction recovery and matrix effect were utilized. Extraction recovery was calculated as the ratio of the ISTDs spiked at the start of the extraction procedure and the ISTDs spiked to the injection solvent prior to MS measurement. This value does not reflect the extraction recovery of metabolites from muscle tissue, but the loss of targeted metabolites during the liquid–liquid extraction process. The matrix effect was calculated by Equation (1), where the ratio of ISTDs is extracted from a muscle sample and a blank sample with only extraction solvents:

Matrix effect = ITSDs extracted form muscle samples ÷ ISTDs extracted from blank samples(1)

For selection of the optimal extraction method, the percentage of the highest extraction recovery for ISTDs for each extraction method was used and calculated by Equation (2):

Percentage (%) = The number of highest recovery in ISTDs ÷ The number of total ISTDs × 100%(2)

For metabolite stability evaluation in mouse muscle tissue, the response ratio was used and obtained by Equation (3):

Response ratio = peak area of the target metabolite ÷ peak area of the assigned ISTD(3)

RStudio (Version 1.4.1106) and R (Version 4.0.5) were used for the statistical analysis of the data statistical—all the figures were made by Graphpad Prism (Version 8.1.1, San Diego, CA, USA).

## 3. Results and Discussion

### 3.1. Development and Evaluation of the Sample Preparation Methods

Four sample preparation methods, i.e., BD, BMC, BMW, BMMW, were systematically compared and evaluated with respect to extraction recovery and matrix effect for a range of metabolite classes by spiking carbon- or deuterium-labelled metabolites (ISTDs) using pig muscle tissue as a surrogate for mouse muscle during method development.

#### 3.1.1. Extraction of Signaling Lipids

**Figure 1:**
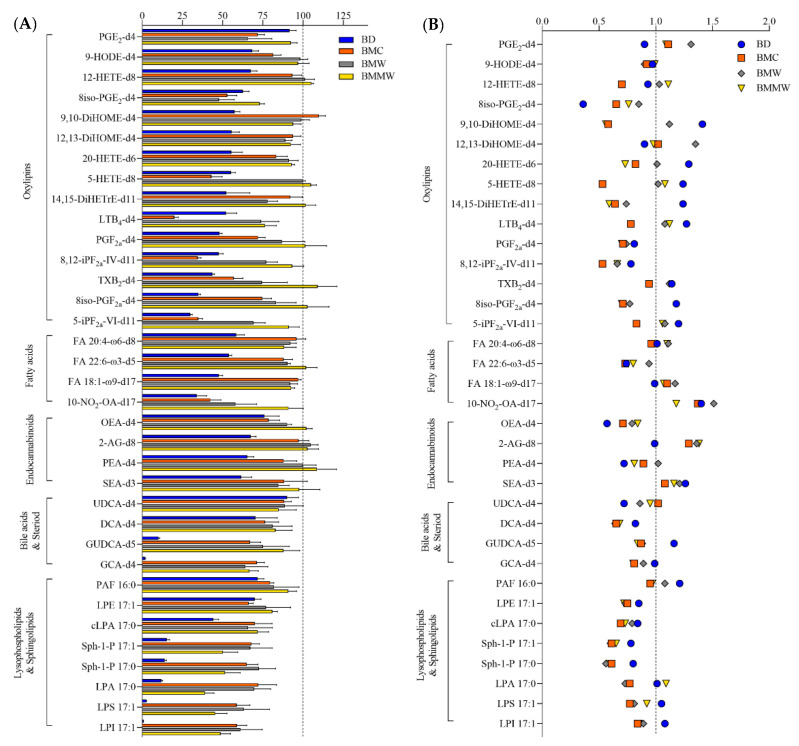
The (**A**) extraction recovery (%) and (**B**) matrix effect of lipids ISTDs by using the four extraction methods: BD, BMC, BMW, and BMMW. Lower recovery values of lysophospholipids and sphingolipids (<91%), and some bile acids, i.e., GCA-d4 (2–71%) and DCA-d4 (70–83%), were observed in all four extraction methods compared to other lipid metabolites. The reason for the lower yield might be because of the less non-polar properties of lysophospholipids and sphingolipids (logP = 2.6–5.4), GCA (logP = 1.4) and DCA (logP = 3.3) than the other classes of lipid metabolites, i.e., fatty acids (logP = 6.0–6.8), endocannabinoids (logP = 5.7–6.7), and oxylipins (logP = 3.1–5.9). The higher recovery of oxylipins reported using the BD method (around 100%) in Alves et al.’s study compared with our BMMW method (>73%) results from the combination of both the organic and aqueous phases for the measurement of these polar lipids [29]. Here, we compared just the organic phase extraction performance for lipid metabolites in the four sample preparation methods.

Five classes of lipid metabolites, i.e., oxylipins, lysophospholipids and sphingolipids, free fatty acids, bile acids and steroids, and endocannabinoids, were analyzed in the organic phase for evaluation of the four extraction methods. Figure 1A showed that the extraction recovery of these lipids using BMC (orange), BMW (brown), and BMMW (yellow) were significantly higher than when using the BD (blue) method. This may be due to the utilization of the more non-polar solvents, MTBE and BuOH (relative polarity is 0.124 and 0.586, respectively [33]), for signaling lipids extraction in BMC, BMW, and BMMW than the two less non-polar solvents, i.e., chloroform and MeOH (relative polarity is 0.259 and 0.762, respectively [33]), in BD. The higher non-polar property contributed to a higher partitioning of all signaling lipids in the organic phase in BMC, BMW, and BMMW. Similar results were also reported in [34], which indicated that lipids in a hydrophobically-associated form can more easily be extracted by relatively non-polar solvents, and the polar solvents, i.e., ethanol and methanol, can disrupt the hydrogen bonding or electrostatic forces between membrane-associated lipids and protein.

To determine the matrix effects of the four extraction methods on the targeted lipid measurements, signals of spiked internal standards in samples with and without muscle tissue were investigated (Equation (1)). For most of the signaling lipids, matrix effect values (Figure 1B) ranged between 0.7–1.4, indicating that there is acceptable impact on MS measurements from the muscle tissue matrix for all four extraction methods.

#### 3.1.2. Extraction of Polar Metabolites

**Figure 2:**
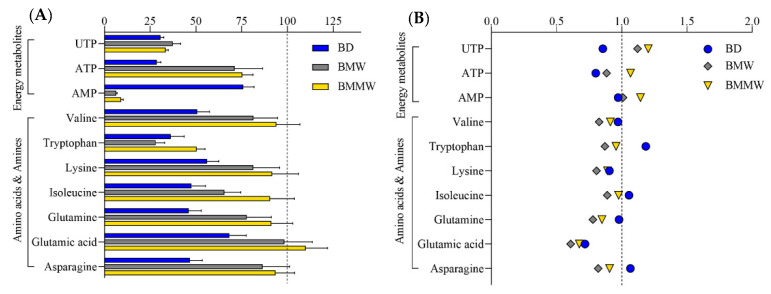
The (**A**) extraction recovery (%) and (**B**) matrix effect of polar ISTDs by using the extraction methods: BD, BMW, and BMMW.

As a non-volatile (citric acid/phosphate) buffer was utilized in the published BMC method, the aqueous phase was rendered unsuitable for the intended LC-MS analysis methods. In addition, the exogenous citric acid affected the analysis of one of our target metabolites, citric acid. Therefore, the aqueous phase of the BMC method was not considered for the polar metabolite analysis. Two separation methods for polar metabolites, i.e., HILIC for central energy metabolites, and CE for amino acids and amines, were used to evaluate the extraction of polar metabolites into the aqueous phase for the three extraction methods (BD, BMW, and BMMW). For amino acids and amines, the extraction recoveries in BMW (brown) and BMMW (yellow) were significantly higher than in BD (blue) (Figure 2A). For energy metabolites, the recovery of ATP and UTP in BMW and BMMW was notably better than in BD; however, the recovery of AMP was dramatically lower compared to BD (Figure 2A). This might be the result of one extra 2-min vortex step with chloroform, water, and MeOH at room temperature in BD, which accelerated the hydrolysis of ATP (or ADP) to AMP. Similar results showing ATP hydrolysis at room temperature were also observed in Becker et al.’s study [35]. Bruno et al. preferred the BD method for polar metabolites in mouse muscle over the MeOH/water extraction method but did not evaluate other methods [36]. Given the stability issues, we concluded that the BD method was not the optimal extraction method for the HILIC measurements of energy metabolites from muscle tissues for our study.

When evaluating the performance of the extraction methods for the CE measurements of amino acids and amines, we note the relatively low recovery obtained for tryptophan (28–50%) as compared to other amino acids and amines. This may be due to its high susceptibility to oxidative degradation [37]—its weakest polar property (logP = −1.1)—and water solubility (1.36 mg/mL) among this class of metabolites (logP ranges from −2.0 to −5.4, water solubility ranges from 80.6 to 210 mg/mL), which contributed to the less passive distribution of tryptophan in the aqueous phase. The weak polar property of UTP (logP = −3.4) may have also contributed to its lower distribution in the aqueous phase compared to ATP (logP = −5.1). The lower extraction recovery of UTP than weakly polar amino acids and amines, i.e., valine (logP = −2.0), may be due to the much higher water solubility of valine (210 mg/mL) than UTP (8.37 mg/mL). Matrix effect values (Figure 2B) were close to one for most of the polar metabolites, demonstrating small impacts from the extraction methods and muscle tissue matrix on MS measurement for the targeted polar metabolites.

#### 3.1.3. Assessment of Sample Preparation Method Yielding Optimal Recovery for Signaling Lipids and Polar Metabolites

**Figure 3:**
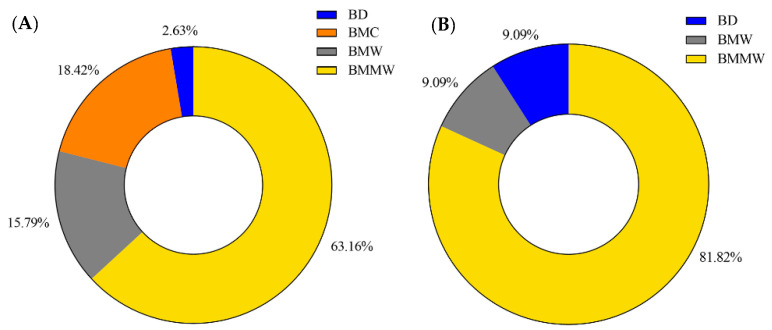
The percentage of the highest extraction recovery each method occupied in (**A**) signaling lipids and (**B**) polar metabolites.

The performance of four extraction methods (BD, BMC, BMW, and BMMW) for signaling lipids and three extraction methods (BD, BMW, and BMMW) for polar metabolites were evaluated and compared by calculating the percentage of the highest extraction recovery for each extraction method for different internal controls for each of the two chemical categories (Equation (2)). The BMMW method turned out to give the best recovery, as deduced from reaching the highest percentage of spiked internal standards for both non-polar (63%) and polar (81%) metabolites (Figure 3), thereby demonstrating that this method resulted in the smallest loss of metabolites during the sample preparation procedure in BMMW for all classes of metabolites of interest. BD was not preferred for mouse muscle extraction not only because of the lower recovery and percentage values, but also due to the rapid hydrolysis observed for ATP (or ADP) to AMP, and the labour required for the reproducible separation of the organic and aqueous phase [24]. Therefore, BMMW was chosen as the extraction method of choice for the targeted non-polar and polar metabolites from small quantities of mouse muscles.

### 3.2. Performance of the Optimal Sample Preparation Method in Mouse Muscle Samples

**Figure 4:**
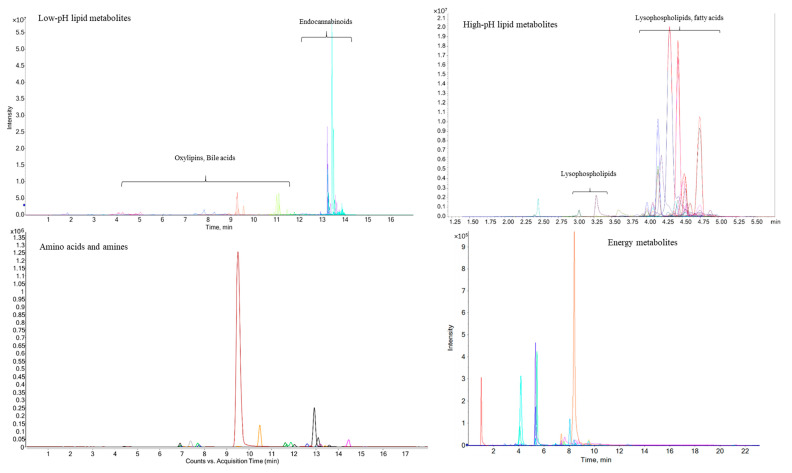
Representative LC/CE-MS chromatograms from different classes of metabolites obtained from *Ercc1^∆/−^* mouse muscle samples.

For the metabolic profiling of mouse muscle, the reported LC-MS and CE-MS detection methods for lipid metabolites [38], energy metabolites [31], and amino acids and amines [32] were utilized. One-hundred-and-nine non-polar and 62 polar targeted metabolites were clearly observed (with a signal to noise ratio > 10) using the LC-MS and CE-MS detection platforms to analyze *Ercc1^∆/−^* mouse muscle tissues (Figure 4). Detailed information of these non-polar (lipid) and polar metabolites for LC-MS and CE-MS analysis is provided in Tables S4 and S5, respectively—in the Supporting Information section. As the sample collection procedure can also influence metabolite stability, the effect of muscle isolation speed on metabolite stability for these targeted metabolites was further investigated.

### 3.3. The Effects of Sample Isolation Speed on Metabolite Stability

For future metabolomics mechanistic studies of sarcopenia, we must utilize a sensitive sample preparation method and a muscle tissue isolation method that preserves metabolite stability. To deduce the effect of sample collection speed on metabolite stability, the response ratios (Equation (3)) of metabolites in fast and delayed muscle tissue isolation were investigated in three muscle specimens, namely the lower hindlimb muscles gastrocnemius and soleus (Gas + Sol), the extensor digitorum longus + tibialis anterior (EDL + TA), and the upper hindlimb muscle quadriceps (Quadr), which are the most commonly used mouse muscles for molecular analyses. In Gas + Sol, significantly higher unsaturated fatty acids (FA18.1-ω9, FA20.3-ω6, FA20.4-ω6, FA20.5-ω3, FA22.4-ω6) and oxylipins (19-20-DiHDPA, 8-9-DiHETrE) were observed in delayed isolation samples compared to the fast isolation (Table 1). These fatty acids and oxylipins are in the arachidonic acid and eicosapentaenoic acid pathways, which are associated with inflammation and age-related diseases [39], and are oxidation sensitive [40,41]. Fifteen-min at room temperature led to longer oxygen exposure and maybe changed the enzymatic activity in these muscle tissues, which contributed to the oxidation and instability of the unsaturated fatty acids, as well as the generation of their downstream metabolites, i.e., oxylipins [42,43]. The higher lysophospholipids (Table 1), i.e., LPE14.0, LPE16.1, LPE20.4, LPE22.4, LPG16.1, LPI20.4, LPI22.4, and LPI22.6, in 15-min delayed isolation muscle tissues might be due to the hydrolysis of the cellular membrane induced by the longer time of oxidation exposure and oxidative damage [44,45], and/or tissue degeneration. The significantly increased pyruvate in Gas + Sol with delayed isolation (Table 2) may be due to the oxidation of lactate [46]. Creatine phosphate is considered to be the “energy pool” in muscle cells and will be preferentially consumed under the condition of insufficient energy and generate its downstream metabolite, creatine [47]. A higher creatine content in Gas + Sol with the 15-min delayed isolation (Table 2) may be explained by the insufficient energy supply in muscle tissues post-dissection and the consumption of creatine phosphate in the muscle cells before the muscle tissue is isolated and snap frozen [47,48]. The Quadr muscle was much more stable than Gas + Sol with 15-min delayed isolation, as only 3 metabolites, i.e., 7-HDoHE, creatine, and PEA, were significantly affected. More altered metabolites were observed in EDL + TA with 15-min delayed isolation compared to both Gas + Sol and Quadr.

The increase in the number of significantly altered metabolites after delayed isolation in the Gas + Sol muscle, compared to Quadr muscle, may be due to the type-I oxidative muscle (soleus) included in Gas + Sol, as well as the type-II glycolytic muscle of Quadr [49,50,51]. The oxidative fibers mainly use aerobic respiration to provide ATP, and glycolytic fibers primarily use anaerobic glycolysis as their energy supply [52], which induced more oxidation in Gas + Sol than Quadr. The largest number of significantly altered metabolites was observed in EDL + TA, which may be due to the varied and unsystematic muscle type composition and fiber density in TA [53,54,55]. Kammoun et. al. found that 57% of type IIB, 3% of hybrid IIAX fibers, and no hybrid IIX/IIB fibers were observed in TA [53]. However, Bloemberg et. al. found mouse white tibialis anterior contained 12.1% hybrid fibers [54]. Lexell et. al. revealed that in TA, the proportion of type-I fibers and fiber density varied significantly but not systematically, and also differed significantly between individuals [55]. Similarly, the fiber types of EDL muscle in *Ercc1^∆/−^* mice are altered in composition compared to normal wild-type controls, having reduced type IIA/IIX and increased type IIB [19]. These variations in TA tissue and/or in mice may have contributed to the observed metabolite alterations in EDL + TA with 15-min delayed isolation at room temperature. The different muscle type proportion may be responsible for the observed differences in the stability of metabolites in the three different kinds of muscle. Given the observed instability of metabolites in muscle tissues with 15-min delayed isolation, fast muscle tissue collection will be preferred for our future sarcopenia study.

## 4. Conclusions

Four extraction methods (BD, BMC, BMW, and BMMW) were compared and evaluated to find the optimal sample preparation method for the simultaneous extraction of targeted non-polar and polar metabolites from a limited amount of muscle tissues. The optimal method, BMMW, had an acceptable matrix effect (close to 1.0) for all metabolites and showed the highest extraction recovery for all types of metabolites, with the best performance of all methods studied for 63% of the signaling lipids and 81% of the polar metabolites. BMMW was used for profiling mouse muscle tissues with quantities as small as 5 mg (dry weight). Our study of sample collection protocols found that fast (<15 min) muscle tissue collection is crucial for metabolite stability. The developed sensitive sample preparation method and fast muscle tissue isolation method will be utilized for future metabolomics mechanistic studies of sarcopenia and animal model studies to evaluate treatments to prevent this syndrome.

## Figures and Tables

**Table 1:** The effect of sample collection speed on lipid metabolites stability in different muscle types (n = 3).

Analytes	Gas + Sol	Quadr	EDL + TA	Analytes	Gas + Sol	Quadr	EDL + TA
FA16.0	ns	ns	*	LEA	*	ns	*
FA18.0	ns	ns	ns	SEA	ns	ns	ns
FA18.1-ω9	*	ns	*	1-AG & 2-AG	*	ns	ns
FA18.3-ω3	ns	ns	ns	CDCA	ns	ns	ns
FA20.3-ω6	*	ns	ns	GCA	ns	ns	ns
FA20.3-ω9	ns	ns	ns	GCDCA	ns	ns	ns
FA20.4-ω6	*	ns	ns	GDCA	ns	ns	ns
FA20.5-ω3	**	ns	ns	GUDCA	ns	ns	ns
FA22.4-ω6	*	ns	*	cLPA16.1	ns	ns	ns
FA22.5-ω3	ns	ns	ns	cLPA18.0	ns	ns	ns
FA22.5-ω6	ns	ns	ns	cLPA18.1	ns	ns	ns
FA22.6-ω3	ns	ns	*	cLPA18.2	ns	ns	ns
10-HDoHE	ns	ns	ns	LPA14.0	ns	ns	ns
11-HDoHE	ns	ns	ns	LPA16.1	ns	ns	ns
11-HETE	ns	ns	ns	LPA18.0	ns	ns	ns
12-13-DiHOME	ns	ns	ns	LPA18.1	ns	ns	ns
12-HEPE	ns	ns	***	LPA18.2	ns	ns	ns
13-14dihydro-15k-PGD2	ns	ns	ns	LPA20.4	ns	ns	*
13-14dihydro-15k-PGE2	ns	ns	*	LPA22.4	ns	ns	ns
13-14dihydro-PGF2α	ns	ns	ns	LPE14.0	*	ns	ns
13-HODE	ns	ns	ns	LPE16.0	ns	ns	ns
14-15-DiHETrE	ns	ns	*	LPE16.1	*	ns	*
14-HDoHE	ns	ns	ns	LPE18.0	ns	ns	ns
8iso-PGE1	ns	ns	*	LPE18.1	ns	ns	*
8iso-PGF1α	ns	ns	ns	LPE18.2	ns	ns	*
15S-HETrE	ns	ns	ns	LPE18.3	ns	ns	*
17-HDoHE	ns	ns	ns	LPE20.3	ns	ns	**
18-HEPE	ns	ns	***	LPE20.4	*	ns	*
19-20-DiHDPA	*	ns	**	LPE20.5	ns	ns	ns
1a-1b-dihomo-PGF2α	ns	ns	ns	LPE22.4	*	ns	*
20-HETE	ns	ns	*	LPE22.5	ns	ns	ns
5-HETE	ns	ns	ns	LPE22.6	ns	ns	*
5-iPF2α-VI	ns	ns	ns	LPG14.0	ns	ns	*
7-HDoHE	ns	*	ns	LPG16.0	ns	ns	ns
8-12-iso-iPF2α-VI	ns	ns	ns	LPG16.1	*	ns	ns
8-9-DiHETrE	*	ns	*	LPG18.0	ns	ns	ns
8-HDoHE	ns	ns	ns	LPG18.1	ns	ns	ns
8-HETE	ns	ns	ns	LPG18.2	ns	ns	ns
8iso-15R-PGF2α	ns	ns	ns	LPG20.3	ns	ns	*
8iso-PGE2	ns	ns	ns	LPG20.4	ns	ns	ns
8iso-PGF2α	ns	ns	ns	LPG22.4	ns	ns	ns
8S-HETrE	ns	ns	ns	LPI16.1	ns	ns	*
9-10-13-TriHOME	ns	ns	ns	LPI18.0	ns	ns	ns
9-10-DiHOME	ns	ns	ns	LPI18.1	ns	ns	ns
9-HEPE	ns	ns	*	LPI18.2	ns	ns	*
9-HETE	ns	ns	ns	LPI20.4	*	ns	*
9-HODE	ns	ns	ns	LPI22.4	*	ns	*
iPF2α-IV	ns	ns	ns	LPI22.6	*	ns	ns
PGD2	ns	ns	ns	LPS18.1	ns	ns	**
PGD3	ns	ns	ns	LPS18.2	ns	ns	**
PGE2	ns	ns	ns	LPS20.4	ns	ns	***
PGF2α	ns	ns	*	LPS22.4	ns	ns	**
TXB2	ns	ns	ns	LPS22.6	*	ns	*
AEA	*	ns	*	OEA	ns	ns	ns
PEA	ns	*	ns				

Note: ns means no significant difference, * means *p* < 0.05, ** means *p* < 0.01, *** means *p* < 0.001. Orange background color means significantly increased; Blue background color means significantly decreased.

**Table 2:** The effect of sample collection speed on stability of energy metabolites, amino acids, and amines in different muscle types (n = 3).

Energy Metabolites							
Analytes	Gas + Sol	Quadr	EDL + TA	Analytes	Gas + Sol	Quadr	EDL + TA
Acetyl-CoA	ns	ns	**	IMP	ns	ns	ns
Adenosine	**	ns	***	Creatine	*	*	ns
ADP	ns	ns	*	Inosine	ns	ns	ns
AMP	ns	ns	ns	α-Ketoglutarate	*	ns	ns
Ascorbic-acid	ns	ns	ns	6-phosphogluconic-acid	ns	ns	*
ATP	ns	ns	**	Malate	ns	ns	ns
cAMP	ns	ns	*	GTP	ns	ns	**
CDP	ns	ns	ns	Guanosine	ns	ns	ns
cis-Aconitate	ns	ns	ns	Oxiglutathione	ns	ns	ns
CMP	ns	ns	ns	Phosphoenolpyruvate	ns	ns	ns
CTP	ns	ns	ns	Pyruvate	**	ns	*
Cytidine	ns	ns	ns	Succinate	ns	ns	*
Dihydroxyacetone-P	ns	ns	*	UDP	ns	ns	
Fructose-6-P	ns	ns	ns	UMP	ns	ns	*
GABA	*	ns	ns	Uridine	ns	ns	**
GDP	ns	ns	ns	UTP	ns	ns	*
Glucose	ns	ns	ns	Xanthine	*	ns	**
Glucose-1-P	ns	ns	ns	Glycerate-3-P	ns	ns	
Glucose-6-P	ns	ns	ns	GMP	ns	ns	**
Glyceraldehyde-3-P	ns	ns	ns	Hypoxanthine	*	ns	ns
**Amino acids and amines**							
Alanine	ns	ns	ns	Methionine	ns	ns	*
Arginine	ns	ns	ns	Phenylalanine	*	ns	ns
Asparagine	ns	ns	ns	Proline	ns	ns	ns
Aspartic-acid	ns	ns	*	Serine	ns	ns	ns
Lysine	ns	ns	ns	Spermidine	ns	ns	ns
Creatinine	ns	ns	ns	Tyrosine	ns	ns	ns
Glutamic-acid	ns	ns	ns	Valine	ns	ns	ns
Glutamine	ns	ns	ns	Threonine	ns	ns	ns
Glycine	ns	ns	*	Ornithine	ns	ns	ns
Histidine	ns	ns	ns	4-Hydroxyproline	*	ns	ns
Leucine	ns	ns	ns	Tryptophan	ns	ns	ns

Note: ns means no significant difference, * means *p* < 0.05, ** means *p* < 0.01, *** means *p* < 0.001. Orange background color means significantly increased; Blue background color means significantly decreased.

## Data Availability

The data presented in this study are accessible through EBI Metabolights repository accession number MTBLS5644 (www.ebi.ac.uk/metabolights/MTBLS5644, accessed on 14 July 2022).

## Supplementary Materials

The following supporting information can be downloaded at: https://www.mdpi.com/article/10.3390/metabo12080742/s1, Table S1: The information of lipid ISTDs; Table S2: The information of amino acids and amines ISTDs; Table S3: The information of energy metabolites ISTDs; Table S4: Detected lipid metabolites in mouse muscle samples; Table S5: Detected polar metabolites in mouse muscle samples.
