# Supplementary material for: A Sample Preparation Method for the Simultaneous Profiling of Signaling Lipids and Polar Metabolites in Small Quantities of Muscle Tissues from a Mouse Model for Sarcopenia
Source: Metabolites. 2022 Aug 12;12(8):742. doi: 10.3390/metabo12080742 (PMC9413361; doi:10.3390/metabo12080742)
Supplement: Supplementary file 1 [file metabolites-12-00742-s001.zip › metabolites-1842839-supplementary/SI/Supporting information.pdf]

Supporting information

A sample preparation method for simultaneous profiling signalling lipids and polar metabolites in small quantities of muscle tissues from a mouse model for sarcopenia

Yupeng He[1], Marlien van Mever[1], Wei Yang[1], Luojiao Huang[1], Rawi Ramautar[1], Yvonne Rijksen[2,3], Wilbert P. Vermeij[2,3], Jan H.J. Hoeijmakers[2,3,4,5], Amy Harms[1], Peter W. Lindenburg[1,6], Thomas Hankemeier[1]*

[1]Metabolomics and Analytics Centre, Leiden Academic Centre for Drug Research, Leiden University, The Netherlands

[2]Princess Máxima Center for Pediatric Oncology, Utrecht, The Netherlands

[3]Oncode Institute, Utrecht, the Netherlands

[4]Department of Molecular Genetics, Erasmus MC Cancer Institute, Erasmus University Medical Center Rotterdam, Rotterdam, The Netherlands

[5]Institute for Genome Stability in Aging and Disease, Cologne Excellence Cluster for Cellular Stress Responses in Aging-Associated Diseases (CECAD), University of Cologne, Cologne, Germany

[6]Research Group Metabolomics, Leiden Center for Applied Bioscience, University of Applied Sciences Leiden, The Netherlands

(*) corresponding author

Einsteinweg 55, 2333 CC Leiden, The Netherlands

Tel.: +31 71 527 1340, E-mail: hankemeier@lacdr.leidenuniv.nl

Table S1. The information of lipid ISTDs.

Name	Concentration (mM)	Precursor Mass (M/Z)	Fragment Mass (M/Z)	Retention Time (min)	Class
Analysed by low pH LC-MS/MS method					
DCA-d4	1.00	395.3	349.1	10.2	Bile Acids & Steroid
GCA-d4	1.00	468.3	74.05	5.5	Bile Acids & Steroid
UDCA-d4	1.00	395.3	395.3	8.1	Bile Acids & Steroid
GUDCA-d5	1.00	453.3	74.1	5	Bile Acids & Steroid
SEA-d3	0.10	331.3	62.2	13.72	Endocannabinoids
OEA-d4	0.10	330.3	66.2	13.35	Endocannabinoids
PEA-d4	0.10	304.3	62.2	13.23	Endocannabinoids
2-AG-d8	0.26	387.3	294.2	13.21	Endocannabinoids
10-NO_2-OA-d17	2.90	343.2	183.2	13.2	Fatty acids
FA 18:1-ω9-d17	3.34	298.1	298.1	13.8	Fatty acids
FA 22:6-ω3-d5	1.50	332.1	288.4	13.4	Fatty acids
FA 20:4-ω6-d8	32.00	311.1	267.2	13.47	Fatty acids
14,15-DiHETrE-d11	0.29	348.2	207.1	9.8	Oxylipins
5-i$PF_{2\alpha}$-VI-d11	0.27	364.2	115.05	3.9	Oxylipins
8,12-i$PF_{2\alpha}$-IV-d11	0.27	364.21	115.05	5.9	Oxylipins
12,13-DiHOME-d4	0.31	317.2	185.1	9.3	Oxylipins
8iso-PGE_2-d4	0.28	355.3	275.25	5.42	Oxylipins
8iso-$PGF_{2\alpha}$-d4	0.28	357.3	197.15	4.75	Oxylipins
9,10-DiHOME-d4	0.31	317.2	203.1	9.5	Oxylipins
9-HODE-d4	0.33	299.2	172.1	11.1	Oxylipins
LTB_4-d4	0.29	339.5	197.1	9.2	Oxylipins
PGE_2-d4	0.28	355.3	275.25	5.42	Oxylipins
$PGF_{2\alpha}$-d4	0.28	357.3	197.15	4.75	Oxylipins
TXB_2-d4	0.27	373.5	173.3	3.7	Oxylipins
20-HETE-d6	0.31	325.2	279.2	10.6	Oxylipins
12-HETE-d8	0.30	327.2	184.1	11.7	Oxylipins
5-HETE-d8	0.30	327.1	116.15	12.1	Oxylipins
Analysed by high pH LC-MS/MS method					
FA 18:1-ω9-d17	3.34	298.2	298.2	4.311	Fatty acids
FA 22:6-ω3-d5	1.50	332.3	288.25	4.107	Fatty acids
cLPA 17:0	2.31	405.2	269.25	5.529	Lysophospholipids & Sphingolipids
PAF 16:0	9.00	572.2	59	6.681	Lysophospholipids & Sphingolipids
LPA 17:0	14.73	423.2	153.05	4.172	Lysophospholipids & Sphingolipids
LPE 17:1	10.73	464.4	267.35	5.352	Lysophospholipids & Sphingolipids
LPI 17:1	0.57	583.4	267.3	4.589	Lysophospholipids & Sphingolipids
LPS 17:1	9.82	508.4	153.2	4.099	Lysophospholipids & Sphingolipids
Sph-1-P 17:0	2.72	366	79.05	3.596	Lysophospholipids & Sphingolipids
Sph-1-P 17:1	2.74	364	79	3.306	Lysophospholipids & Sphingolipids

Table S2. The information of amino acids and amines ISTDs.

Name	Concentration (mg/mL)	Molar mass (M/Z)	Retention Time (min)	Class
Analysed by HILIC-MS method				
Asparagine-$^{13}C_4$, $^{15}N_2$	0.5	137.0537	12.84	Amine
Glutamate-$^{13}C_5$, d_5, ^{15}N	0.5	157.0911	8.38	Amino acid
Isoleucine-^{13}C, ^{15}N	0.5	132.0877	7.93	Amino acid
Valine-$^{13}C_5$	0.5	121.0885	6.89	Amino acid
Leucine-d_3	0.5	133.1062	7.64	Amino acid
Analysed by CE-MS method				
Aspartate-$^{13}C_4$, d_3, ^{15}N	0.5	142.0448	14.10	Amino acid
Glutamate-$^{13}C_5$, d_5, ^{15}N	0.5	159.0604	12.80	Amino acid
Glutamine-$^{13}C_5$	0.5	152.0764	12.70	Amine
Glycine-d_2	0.5	78.0322	9.80	Amino acid
Isoleucine-^{13}C, ^{15}N	0.5	134.0877	11.02	Amino acid
Tryptophan-$^{13}C_{11}$, $^{15}N_2$	0.5	218.0972	12.50	Amino acid
Valine-$^{13}C_5$	0.5	123.0863	11.40	Amino acid

Table S3. The information of energy metabolites ISTDs.

Name	Concentration	Molar mass (M/Z)	Retention Time (min)	Class
Spiking before sample extraction (for sample extraction method evaluation)				
ATP-$^{13}C_{10}$, $^{15}N_5$	10 mg/mL	520.9885	12.74	Energy metabolites
AMP-$^{13}C_{10}$, $^{15}N_5$	10 mg/mL	361.0558	7.07	Energy metabolites
UTP-$^{13}C_9$, $^{15}N_2$	10 mg/mL	493.9613	13.90	Energy metabolites
Spiking before MS analysis (only for response ratio calculation)				
Fumarate-d_2	500 mM	117.0162	13.10	Energy metabolites
Pyruvate-$^{13}C_3$	500 mM	90.0188	6.45	Energy metabolites
Succinate-d_4	500 mM	121.0444	7.95	Energy metabolites
UMP-$^{15}N_2$	500 mM	325.0227	7.10	Energy metabolites

Table S4. Detected lipid metabolites in mouse muscle samples.

Name	Precursor Mass (M/Z)	Fragment Mass (M/Z)	Retention Time (min)	ChEBI ID	ISTD	Class
Analysed by low pH LC-MS/MS method						
CDCA	391.2	373.25	10	16755	DCA-d4	Bile Acids & Steroid
GCA	464.2	74.1	5.5	17687	GCA-d4	Bile Acids & Steroid
GCDCA	448.21	74	8.3	36274	GUDCA-d5	Bile Acids & Steroid
GDCA	448.22	74	8.7	27471	GUDCA-d5	Bile Acids & Steroid
GUDCA	448.2	74	5	89929	GUDCA-d5	Bile Acids & Steroid
1-AG and 2-AG *(two peaks merged to one)*	379.21	287	13.3	34071 and 52392	2-AG-d8	Endocannabinoids
AEA	348	62	13	2700	SEA-d3	Endocannabinoids
LEA	324	62	12.9	64032	SEA-d3	Endocannabinoids
OEA	326	62	13.4	71466	OEA-d4	Endocannabinoids
PEA	300	62	13.2	71464	PEA-d4	Endocannabinoids
SEA	328	62	13.7	85299	SEA-d3	Endocannabinoids
FA16.0	255.2	237.2	13.8	15756	FA 18:1-ω9-d17	Fatty acids
FA18.0	283.2	265.2	14.1	28842	FA 18:1-ω9-d17	Fatty acids
FA18.1-ω9	281.1	263.2	13.8	16196	FA 18:1-ω9-d17	Fatty acids
FA20.3-ω6	305.1	261	13.7	NA	FA 20:4-ω6-d8	Fatty acids
FA20.3-ω9	305.1	261	13.8	72865	FA 20:4-ω6-d8	Fatty acids
FA20.4-ω6	303	259	13.5	15843	FA 20:4-ω6-d8	Fatty acids
FA20.5-ω3	301.1	257.2	13.3	28364	FA 20:4-ω6-d8	Fatty acids
FA22.6-ω3	327.1	283.1	13.4	28125	FA 22:6-ω3-d5	Fatty acids
10-HDoHE	343.21	153.1	11.8	72640	12-HETE-d8	Oxylipins
11-HDoHE	343.2	121.1	11.9	72794	12-HETE-d8	Oxylipins
11-HETE	319.2	167.1	11.6	72606	12-HETE-d8	Oxylipins
12,13-DiHOME	313.2	183.1	9.3	72665	12,13-DiHOME-d4	Oxylipins
12-HEPE	317.2	179.1	10.8	NA	12-HETE-d8	Oxylipins
13,14dihydro-15k-PGD_2	351.21	175.1	6.6	72603	PGE_2-d4	Oxylipins
13,14dihydro-15k-PGE_2	351.2	175.1	5.7	15550	PGE_2-d4	Oxylipins
13,14dihydro-$PGF_{2\alpha}$	355.2	311.3	5.4	63976	$PGF_{2\alpha}$-d4	Oxylipins
13-HODE	295.2	195.2	11	72639	9-HODE-d4	Oxylipins
14,15-DiHETrE	337.2	207.1	9.8	63966	14,15-DiHETrE-d11	Oxylipins
14-HDoHE	343.2	205.1	11.7	72647	12-HETE-d8	Oxylipins
15S-HETrE	321.2	221.1	12	88348	5-HETE-d8	Oxylipins
17-HDoHE	343.2	281.2	11.2	72637	12-HETE-d8	Oxylipins
18-HEPE	317.2	299.2	10.4	72802	12-HETE-d8	Oxylipins
19,20-DiHDPA	361.2	273.2	9.9	72657	14,15-DiHETrE-d11	Oxylipins
1a,1b-dihomo-$PGF_{2\alpha}$	381.1	337.45	7.3	NA	$PGF_{2\alpha}$-d4	Oxylipins
20-HETE	319.2	289.2	10.6	34306	20-HETE-d6	Oxylipins
5-HETE	319.2	115.15	12.3	28209	5-HETE-d8	Oxylipins
5-i$PF_{2\alpha}$-VI	353.2	115.05	4	140933	5-i$PF_{2\alpha}$-VI-d11	Oxylipins
7-HDoHE	343.2	281.2	12.1	72623	12-HETE-d8	Oxylipins

8-12-iso-iPF$_{2\alpha}$-VI	353.21	115.05	6	NA	8,12-iPF$_{2\alpha}$-IV-d11	Oxylipins
8-9-DiHETrE	337.2	127.2	10.3	63970	14,15-DiHETrE-d11	Oxylipins
8-HDoHE	343.2	189.1	12.1	72610	12-HETE-d8	Oxylipins
8-HETE	319.2	155.1	11.8	34486	5-HETE-d8	Oxylipins
8iso-15R-PGF$_{2\alpha}$	353.1	193.1	3.5	NA	8iso-PGF$_{2\alpha}$-d4	Oxylipins
8iso-PGE$_1$	353.2	317.2	4.7	NA	8iso-PGE$_2$-d4	Oxylipins
8iso-PGE$_2$	351.1	271.15	4.5	131888	8iso-PGE$_2$-d4	Oxylipins
8iso-PGF$_{1\alpha}$	355.2	311.1	4.5	NA	8iso-PGF$_{2\alpha}$-d4	Oxylipins
8iso-PGF$_{2\alpha}$	353.1	193.1	3.7	34509	8iso-PGF$_{2\alpha}$-d4	Oxylipins
8(S)-HETrE	321.2	303.2	12.3	140473	12-HETE-d8	Oxylipins
9-10-13-TriHOME	329.2	171.1	4.4	NA	12-HETE-d8	Oxylipins
9-10-DiHOME	313.2	201.05	9.6	72663	9,10-DiHOME-d4	Oxylipins
9-HEPE	317.2	167.25	10.9	89570	12-HETE-d8	Oxylipins
9-HETE	319.21	167.1	12	72786	12-HETE-d8	Oxylipins
9-HODE	295.21	171.1	11.1	72651	9-HODE-d4	Oxylipins
iPF$_{2\alpha}$-IV	353.3	127.1	3.2	NA	5-iPF$_{2\alpha}$-VI-d11	Oxylipins
PGD$_2$	351.1	271.15	5.1	15555	PGE$_2$-d4	Oxylipins
PGD$_3$	349.2	269.2	3.6	34939	PGE$_2$-d4	Oxylipins
PGE$_2$	351.1	271.15	4.8	15551	PGE$_2$-d4	Oxylipins
PGF$_{2\alpha}$	353.1	193.1	4.6	28031	PGF$_{2\alpha}$-d4	Oxylipins
TXB$_2$	369.2	169.1	3.8	15553	TXB$_2$-d4	Oxylipins
Analysed by high pH LC-MS/MS method						
FA18.3-ω3	277.1	233.15	3.9	27432	FA 18:1-ω9-d17_	Fatty acids
FA22.4-ω6	331.2	287.4	5.1	NA	FA 22:6-ω3-d5	Fatty acids
FA22.5-ω3	329.2	285.4	4.7	NA	FA 22:6-ω3-d5	Fatty acids
FA22.5-ω6	329.2	285.4	4.8	NA	FA 22:6-ω3-d5	Fatty acids
cLPA16.1	389.1	253	4.7	NA	cLPA 17:0	Lysophospholipids & Sphingolipids
cLPA18.0	419.1	283	5.9	NA	cLPA 17:0	Lysophospholipids & Sphingolipids
cLPA18.1	417.2	281	5.4	62838	cLPA 17:0	Lysophospholipids & Sphingolipids
cLPA18.2	415.1	279	5	NA	cLPA 17:0	Lysophospholipids & Sphingolipids
LPA14.0	381.2	153.05	3.2	62833	LPA 17:0	Lysophospholipids & Sphingolipids
LPA16.1	407.2	153.05	3.5	75070	LPA 17:0	Lysophospholipids & Sphingolipids
LPA18.0	437.3	153.05	4.5	74850	LPA 17:0	Lysophospholipids & Sphingolipids
LPA18.1	435.2	153.05	4.1	62837	LPA 17:0	Lysophospholipids & Sphingolipids
LPA18.2	433.2	153.05	3.7	62834	LPA 17:0	Lysophospholipids & Sphingolipids
LPA20.4	457	153.05	3.9	73792	LPA 17:0	Lysophospholipids & Sphingolipids
LPA22.4	485.2	153.05	4.4	NA	LPA 17:0	Lysophospholipids & Sphingolipids
LPE14.0	424.4	196.15	4.7	NA	LPE 17:1	Lysophospholipids & Sphingolipids
LPE16.0	452.4	196.15	5.5	73134	LPE 17:1	Lysophospholipids & Sphingolipids

LPE16.1	450.4	196.15	5	NA	LPE 17:1	Lysophospholipids & Sphingolipids
LPE18.0	480.4	196.25	6.3	83047	LPE 17:1	Lysophospholipids & Sphingolipids
LPE18.1	478.4	196.25	5.7	75168	LPE 17:1	Lysophospholipids & Sphingolipids
LPE18.2	476.4	196.25	5.2	83058	LPE 17:1	Lysophospholipids & Sphingolipids
LPE18.3	474.4	196.25	4.9	NA	LPE 17:1	Lysophospholipids & Sphingolipids
LPE20.3	502.4	196.15	5.6	NA	LPE 17:1	Lysophospholipids & Sphingolipids
LPE20.4	500.4	196.15	5.3	64395	LPE 17:1	Lysophospholipids & Sphingolipids
LPE20.5	498.4	196.15	4.9	NA	LPE 17:1	Lysophospholipids & Sphingolipids
LPE22.4	528.4	196.15	5.9	NA	LPE 17:1	Lysophospholipids & Sphingolipids
LPE22.5	526.4	196.15	5.5	NA	LPE 17:1	Lysophospholipids & Sphingolipids
LPE22.6	524.4	196.15	5.3	72747	LPE 17:1	Lysophospholipids & Sphingolipids
LPG14.0	455.1	227.3	4.1	73092	LPI 17:1	Lysophospholipids & Sphingolipids
LPG16.0	483.1	255.3	4.9	75376	LPI 17:1	Lysophospholipids & Sphingolipids
LPG16.1	481.1	253.3	4.3	138795	LPI 17:1	Lysophospholipids & Sphingolipids
LPG18.0	511.1	283.3	5.5	73091	LPI 17:1	Lysophospholipids & Sphingolipids
LPG18.1	509.1	281.3	5	72952	LPI 17:1	Lysophospholipids & Sphingolipids
LPG18.2	507.1	279.3	4.6	NA	LPI 17:1	Lysophospholipids & Sphingolipids
LPG20.3	533.1	305.3	5	NA	LPI 17:1	Lysophospholipids & Sphingolipids
LPG20.4	531.1	303.3	4.8	NA	LPI 17:1	Lysophospholipids & Sphingolipids
LPG22.4	559.1	331.3	5.2	NA	LPI 17:1	Lysophospholipids & Sphingolipids
LPI16.1	569.1	253.25	4.3	NA	LPI 17:1	Lysophospholipids & Sphingolipids
LPI18.0	599.1	283.25	5.4	NA	LPI 17:1	Lysophospholipids & Sphingolipids
LPI18.1	597.1	281.25	4.8	NA	LPI 17:1	Lysophospholipids & Sphingolipids
LPI18.2	595.1	153.05	4.5	NA	LPI 17:1	Lysophospholipids & Sphingolipids
LPI20.4	619.1	303.25	4.6	NA	LPI 17:1	Lysophospholipids & Sphingolipids
LPI22.4	647.2	331.2	5.1	NA	LPI 17:1	Lysophospholipids & Sphingolipids
LPI22.6	643.2	327.2	4.6	138564	LPI 17:1	Lysophospholipids & Sphingolipids
LPS18.1	522.4	153.1	4.4	52649	LPS 17:1	Lysophospholipids & Sphingolipids
LPS18.2	520.1	153.05	4.1	NA	LPS 17:1	Lysophospholipids & Sphingolipids
LPS20.4	544.1	153.05	4.1	85435	LPS 17:1	Lysophospholipids & Sphingolipids

LPS22.4	572.1	153.05	4.6	NA	LPS 17:1	Lysophospholipids & Sphingolipids
LPS22.6	568.1	153.05	4.1	NA	LPS 17:1	Lysophospholipids & Sphingolipids

(Note: NA means nothing found).

Table S5. Detected polar metabolites in mouse muscle samples.

**Metabolites name**	**ChEBI ID**	**Molar mass (M/Z)**	**Retention time (min)**	**ISTD**	**Class**
Analysed by HILIC-MS method (negative ionization mode)					
6-phosphogluconic acid	48928	275.0174	10.1	Fumarate-d_2	Energy metabolites
Acetyl-CoA	15351	808.1185	7.4	Fumarate-d_2	Energy metabolites
Adenosine	16335	266.0889	3.8	Pyruvate-^{13}C3	Energy metabolites
ADP	16761	426.0221	9.4	AMP-$^{13}C_{10}$,$^{15}N_5$	Energy metabolites
AMP	16027	346.0558	7.0	AMP-$^{13}C_{10}$,$^{15}N_5$	Energy metabolites
Ascorbic acid	29073	175.0242	6.7	Valine-$^{13}C_5$	Energy metabolites
ATP	15422	505.9885	11.5	ATP-$^{13}C_{10}$,$^{15}N_5$	Energy metabolites
cAMP	17489	328.0452	5.7	UMP-$^{15}N_2$	Energy metabolites
CDP	17239	402.0109	10.7	UMP-$^{15}N_2$	Energy metabolites
cis-Aconitate	16383	173.0085	6.7	Valine-$^{13}C_5$	Energy metabolites
CMP	17361	322.0446	8.8	UMP-$^{15}N_2$	Energy metabolites
CTP	17677	481.9772	13.0	UMP-$^{15}N_2$	Energy metabolites
Cytidine	17562	242.0776	5.7	Leucine-d_3	Energy metabolites
Dihydroxyacetone-P	16108	168.9907	9.1	Fumarate-d_2	Energy metabolites
Fructose-6-P	78697	259.0224	9.6	Fumarate-d_2	Energy metabolites
GABA	16865	102.0561	10.6	Asparagine-$^{13}C_4$,$^{15}N_2$	Amino acid
GDP	17552	442.0171	13.3	UMP-$^{15}N_2$	Energy metabolites
Glucose	17234	179.0561	7.5	Fumarate-d_2	Energy metabolites
Glucose-1-P	58601	259.0224	9.1	Fumarate-d_2	Energy metabolites
Glucose-6-P	14314	259.0224	10.2	Fumarate-d_2	Energy metabolites
Glyceraldehyde-3-P	17138	168.9907	8.4	Fumarate-d_2	Energy metabolites
Glycerate-3-P	58272	184.9857	9.2	Fumarate-d_2	Energy metabolites
GMP	NA	362.0507	8.9	UMP-$^{15}N_2$	Energy metabolites
GTP	15996	521.9834	13.6	UMP-$^{15}N_2$	Energy metabolites
Guanosine	16750	282.0838	6.1	Isoleucine-^{13}C, ^{15}N	Energy metabolites
Hypoxanthine	17368	135.0306	4.1	Pyruvate-$^{13}C_3$	Energy metabolites
IMP	17202	347.0398	7.9	UMP-$^{15}N_2$	Energy metabolites
Inosine	17596	267.0728	5.4	Leucine-d_3	Energy metabolites
Malate	25115	133.0142	7.1	Fumarate-d_2	Energy metabolites
Oxiglutathione	167606	611.1441	11.8	Asparagine-$^{13}C_4$, $^{15}N_2$	Energy metabolites
Phosphoenolpyruvate	18021	166.9751	8.9	Asparagine-$^{13}C_4$, $^{15}N_2$	Energy metabolites
Pyruvate	15361	87.0088	3.7	Pyruvate-$^{13}C_3$	Energy metabolites
Succinic acid	15741	117.0193	5.8	Succinate-d_4	Energy metabolites
UDP	17659	402.9949	9.6	UMP-$^{15}N_2$	Energy metabolites
UMP	28895	323.0286	7.2	UMP-$^{15}N_2$	Energy metabolites
Uridine	16704	243.0616	4.3	Pyruvate-$^{13}C_3$	Energy metabolites

UTP	15713	482.9613	11.6	UTP-$^{13}C_9$, $^{15}N_2$	Energy metabolites
Xanthine	15318	151.0255	3.9	Pyruvate-$^{13}C_3$	Energy metabolites
α-Ketoglutarate	80619	145.0142	6.7	Fumarate-d_2	Energy metabolites
Tyrosine	18186	180.0666	7.4	Valine-$^{13}C_5$	Amino acid
Alanine	15570	90.0550	8.9	Glutamate-$^{13}C_5$, d_5, ^{15}N	Amino acid
Phenylalanine	28044	164.0717	5.6	Leucine-d_3	Amino acid
Asparagine	17196	131.0462	9.8	Asparagine-$^{13}C_4$, $^{15}N_2$	Amine
Leucine	25017	130.0874	5.8	Leucine-d_3	Amino acid
Ornithine	18257	131.0826	10.5	Asparagine-$^{13}C_4$, $^{15}N_2$	Amino acid
Analysed by CE-MS method (positive ionization mode)					
Creatine	16919	132.0768	9.6	Glycine-d_2	Energy metabolites
Arginine	29016	175.1190	7.6	Valine-$^{13}C_5$	Amino acid
Spermidine	16610	146.1652	4.5	Glutamine-$^{13}C_5$	Amine
Aspartic acid	17364	134.0448	14.1	Aspartate-$^{13}C_4$, d_3, ^{15}N	Amino acid
Lysine	18019	147.1190	7.2	Valine-$^{13}C_5$	Amino acid
Valine	27266	118.0863	11.4	Valine-$^{13}C_5$	Amino acid
Methionine	16811	150.0583	12.8	Isoleucine-^{13}C, ^{15}N	Amino acid
Glutamine	28300	147.0764	12.7	Glutamine-$^{13}C_5$	Amine
Serine	17822	106.0499	11.8	Aspartate-$^{13}C_4$, d_3, ^{15}N	Amino acid
Threonine	16857	120.0655	12.3	Aspartate-$^{13}C_4$, d_3, ^{15}N	Amino acid
Glutamic acid	18237	148.0604	12.8	Glutamate-$^{13}C_5$, d_5, ^{15}N	Amino acid
Glycine	15428	76.0393	9.8	Glycine-d_2	Amino acid
Histidine	27570	156.0768	7.7	Valine-$^{13}C_5$	Amino acid
Tryptophan	16828	205.0972	12.5	Tryptophan-$^{13}C_{11}$, $^{15}N_2$	Amino acid
4-Hydroxyproline	20392	132.0655	9.3	Valine-$^{13}C_5$	Amino acid
Proline	26271	116.0706	13.3	Valine-$^{13}C_5$	Amino acid
Creatinine	16737	114.0662	6.8	Glycine-d_2	Amine

(Note: NA means nothing found).
